# Genomic expression profiling of mature soybean (*Glycine max*) pollen

**DOI:** 10.1186/1471-2229-9-25

**Published:** 2009-03-06

**Authors:** Farzad Haerizadeh, Chui E Wong, Prem L Bhalla, Peter M Gresshoff, Mohan B Singh

**Affiliations:** 1 Plant Molecular Biology and Biotechnology Laboratory, ARC Centre of Excellence for Integrative Legume Research, Faculty of Land and Food resources, The University of Melbourne, Parkville 3010, Australia; 2 ARC Centre of Excellence for Integrative Legume Research, The University of Queensland, Brisbane, Australia

## Abstract

**Background:**

Pollen, the male partner in the reproduction of flowering plants, comprises either two or three cells at maturity. The current knowledge of the pollen transcriptome is limited to the model plant systems *Arabidopsis thaliana *and *Oryza sativa *which have tri-cellular pollen grains at maturity. Comparative studies on pollen of other genera, particularly crop plants, are needed to understand the pollen gene networks that are subject to functional and evolutionary conservation. In this study, we used the Affymetrix Soybean GeneChip^® ^to perform transcriptional profiling on mature bi-cellular soybean pollen.

**Results:**

Compared to the sporophyte transcriptome, the soybean pollen transcriptome revealed a restricted and unique repertoire of genes, with a significantly greater proportion of specifically expressed genes than is found in the sporophyte tissue. Comparative analysis shows that, among the 37,500 soybean transcripts addressed in this study, 10,299 transcripts (27.46%) are expressed in pollen. Of the pollen-expressed sequences, about 9,489 (92.13%) are also expressed in sporophytic tissues, and 810 (7.87%) are selectively expressed in pollen. Overall, the soybean pollen transcriptome shows an enrichment of transcription factors (mostly zinc finger family proteins), signal recognition receptors, transporters, heat shock-related proteins and members of the ubiquitin proteasome proteolytic pathway.

**Conclusion:**

This is the first report of a soybean pollen transcriptional profile. These data extend our current knowledge regarding regulatory pathways that govern the gene regulation and development of pollen. A comparison between transcription factors up-regulated in soybean and those in *Arabidopsis *revealed some divergence in the numbers and kinds of regulatory proteins expressed in both species.

## Background

In flowering plants, pollen development occurs in the anthers. The meiotic division of diploid sporogenous cells gives rise to a tetrad of haploid microspores. The microspores then undergo an asymmetric mitotic division, giving rise to a smaller generative cell enveloped within a larger vegetative cell [1]. The generative cell divides once again to give rise to the two haploid sperm cells required for double fertilization. In most plants, the pollen is bi-cellular at anther dehiscence, with the division of generative cells taking place during pollen tube growth in the female tissues. However, in some cases such as crucifers and grasses, this division takes place while the pollen is still undergoing maturation in the anther.

In the last decade, the knowledge of pollen transcriptome has emerged with the development of large-scale transcriptional profiling techniques. This is exemplified by a number of studies carried out using model species such as *Arabidopsis thaliana *[2-5] or *Oryza sativa *with a recent report on allergen transcripts [6]. Studies on *Arabidopsis *pollen transcriptome showed that 9.7% of the 13,977 pollen-expressed mRNAs were selectively expressed in pollen; among them, many genes had an unknown function or were reported to be functionally associated with signalling pathways and cell wall metabolism [4]. These studies also revealed differences among the cell cycle regulators, cytoskeleton genes, and signalling in pollen as compared to sporophytic tissues [2-5].

The current knowledge of the pollen transcriptome however, is limited to *Arabidopsis *and rice that have tri-cellular pollen grains at maturity. Comparative studies on pollen of other genera, particularly legume crop plants, are needed to understand the pollen gene networks that are subjected to functional and evolutionary conservation. In this study, we present the transcript profile of the mature soybean pollen that is bi-cellular as compared to sporophytic tissues assayed on the soybean GeneChip^®^. Among the transcripts identified to be up-regulated in the pollen in comparison to the sporophytic tissues, we observed many that are unknown as well as transcripts with putative annotation. That has allowed us to infer pollen regulatory roles for various families of transcription factors as well as products associated with protein destination and storage, signal transduction, transporters and heat shock-associated proteins. The data presented here represent a rich source of novel target genes for further studies into molecular processes that govern the development of pollen.

## Results and discussion

### Detection of differentially expressed transcripts in soybean mature pollen

Using the soybean GeneChip^®^, we compared the transcript profiles of soybean pollen with that of sporophytic tissues consisting of an equal mix of RNA derived from leaves and stems of 10-day-old soybean seedlings. The raw intensity data generated from the microarray hybridization experiment were imported into AffylmGUI [7] and were analysed as outlined in Materials and Methods.

**Figure 1:**
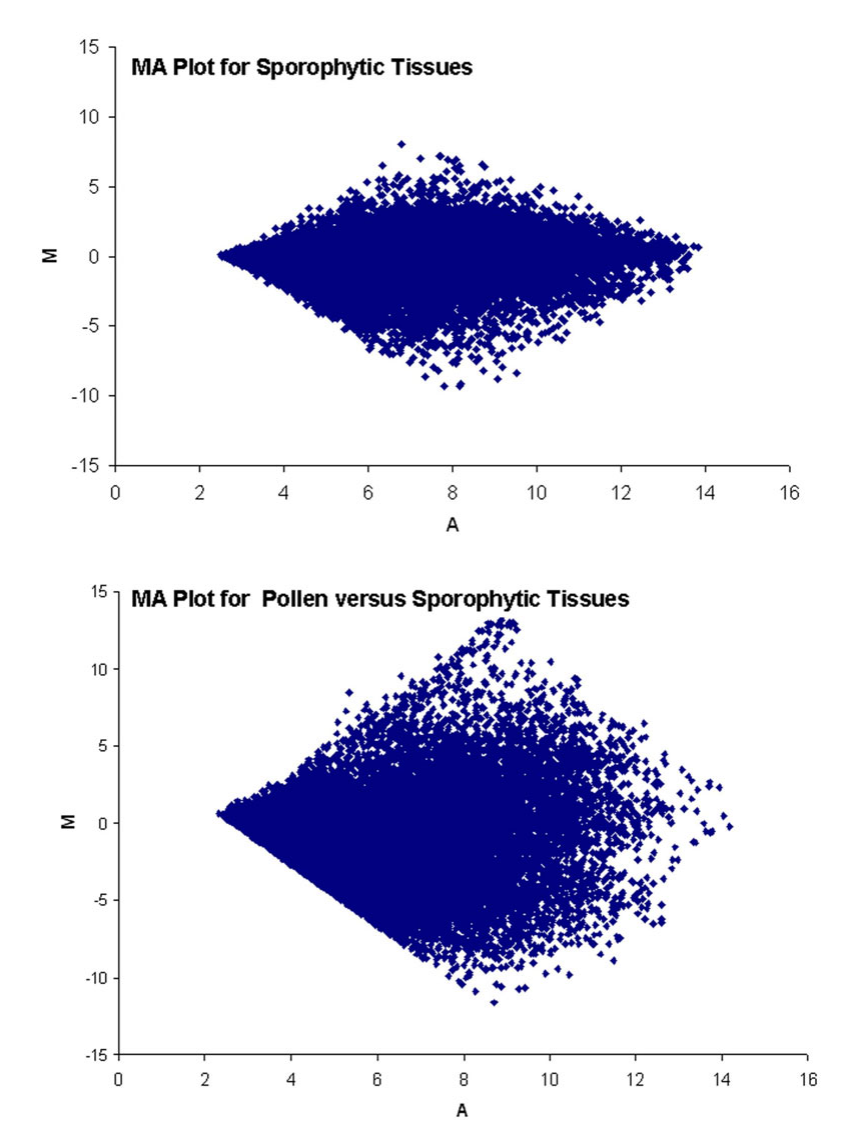
**MA plot comparing the transcript profile of pollen against sporophytic tissues (stems, roots and leaves tissues) or shoot apical meristems (SAM; Haerizadeh et al, unpublished) against stems, roots and leaves tissues (this study)**.

When the normalized data were visually displayed by scatter-plotting the log_2_-transformed signal intensities of the two different samples, there was much complexity and differences on the transcript pattern between pollen and sporophytic tissues as indicated by the greater scatter of the points in the plot in comparison to a similar plot between sporophytic tissues [i.e.] stems, roots and leaves (this study) versus shoot apical meristem (Haerizadeh et al., unpublished) (Figure 1).

The soybean GeneChip^® ^used contains probe sets for 37,500 transcripts and the resulting analysis revealed that approximately 27% of these are expressed in the soybean pollen while 75% are being expressed in sporophytic tissues. This difference reflects the specialization of pollen as compared to other tissues with respect to providing a specific set of transcripts for specific functions such as germination, pollen tube growth, and the subsequent process of fertilization. Meanwhile, only 7.87% of the pollen-expressed genes are likely to be pollen-specific as no 'present' calls were detected for the corresponding probe sets in the sporophytic tissues. A total of 8,763 transcripts show statistically significant differential regulation in pollen as compared to sporophytic tissues with 1,686 of them showing higher expression levels in the pollen than the sporophytic tissues (at adjusted p-value < 0.05; Additional File 1 and Additional File 2). When the expression pattern for sporophytic tissue-expressed chlorophyll a/b binding protein family members were examined, none of these transcripts were represented in the pollen-expressed dataset and hence validate our experimental approach.

### Functional categories of transcripts differentially expressed in pollen

**Figure 2:**
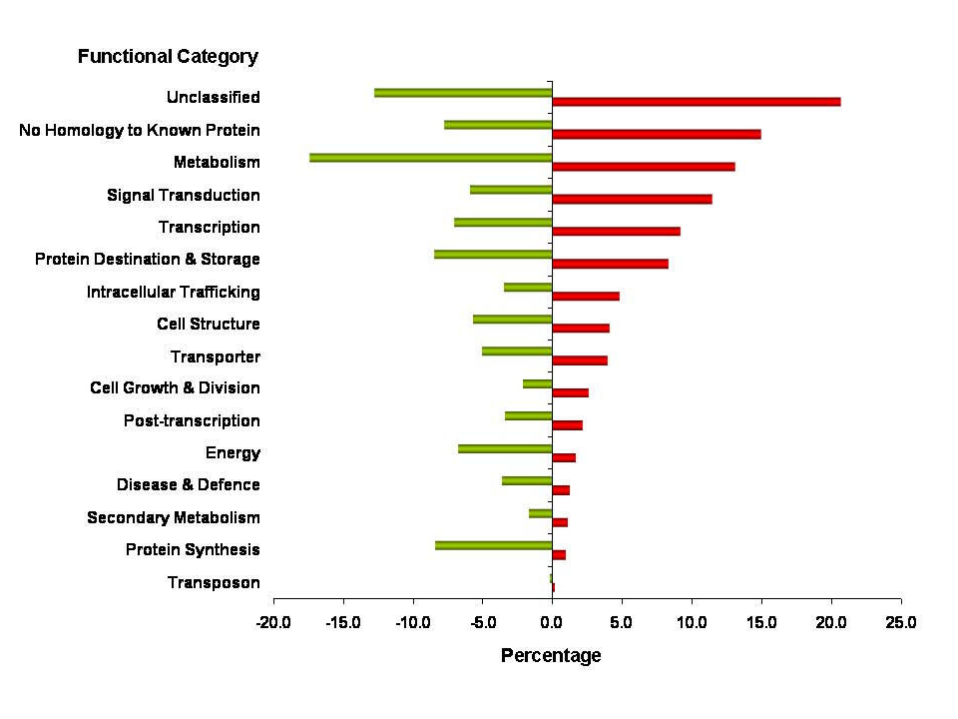
**Functional categorization of up- and down-regulated transcripts in the soybean mature pollen in comparison to sporophytic tissues**. Red or Green bar denotes up-or down-regulated categories, respectively

The transcripts represented by the soybean GeneChip^® ^have been annoated as described in Materials and Methods. This allowed us to examine functional categories of transcripts that are up- or down-regulated in the pollen. As shown in Figure 2, although many of the genes fall into "unclassified" or "no homology to known protein" categories, the general distribution and over-representation of categories such as intracellular trafficking, signal transduction and transcription are evident. The up-regulated transcripts in the "no homology to known protein" category provide a valuable opportunity for the initiation of many functional analysis experiments toward an in-depth understanding of the pollen gene regulatory system and its components, which are presently incomplete.

It is interesting to note that none of the significantly up-regulated transcripts encode products that are related to the small RNA pathways (Additional File 1). A closer inspection of the expression values revealed that all of the small RNA pathways associated transcripts have signals below the detection threshold. This is consistent with a previous report [3] although a recent study by the same group has revealed the detection of 3 out of 15 genes of the ARGONAUTE family that were previously below the detection limit. The authors have attributed this discrepancy to "improved chemistry for sample processing, array hybridization, and staining that resulted in a better signal to noise ratio and thus a higher sensitivity" [8]. It is equally likely that the small RNA pathways are only active in the generative cells and hence further transcript profiling work on gametes shall resolve this issue.

### Top 30 candidates up-regulated in the pollen

The top 30 most highly up-regulated transcripts in pollen in comparison to sporophytic tissues are those predicted to encode cell wall-related proteins such as pectate lyase and pectin esterase family proteins, rapid alkalinization factor (RALF), multi-copper oxidase, and some transporters, along with unknown and novel genes (Table 1). RALF, a 5 kDa ubiquitous polypeptide in plants was first reported as RALF gene in tobacco encoding a ubiquitous 115-amino acid protein, which is processed into a 5-kD signaling peptide [9]. The peptide induced a rapid alkalinization of the culture medium of tobacco suspension-cultured cells and a concomitant activation of an intracellular mitogen-activated protein kinase [9]. RALF is considered as a potential signaling molecule and a putative RALF receptor has been detected in plasma membranes [10]. RALF-LIKE 10 is selectively expressed in Arabidopsis pollen [5]. In our data on soybean pollen two RALF isoforms, RALF-Like 11 and RALF-LIKE 19 show selective expression in pollen. The conserved up-regulation of genes encoding RALF-like signaling peptides in soybean and *Arabidopsis *pollen implicates its essential role in pollen development. However, further experiments involving gain-of-function or loss-of-function mutants are required to address this hypothesis.

**Table 1:** Top 30 up-regulated transcripts in soybean pollen in comparison to sporophytic tissues.

**Affymetrix Probe ID**	**Log_2 _Ratio**	**Annotation**
GmaAffx.9455.1.S1_at	11.0	pectate lyase family protein
GmaAffx.71146.1.S1_at	10.9	pectinesterase family protein
GmaAffx.79807.1.S1_at	10.8	No BLASTX match
GmaAffx.64699.1.S1_at	10.6	Rapid alkalinization factor 11 (RALF-LIKE 11)
GmaAffx.58015.1.S1_at	10.6	Rapid alkalinization factor 19 (RALF-LIKE 19)
GmaAffx.66571.1.S1_at	10.5	pectinesterase family protein
GmaAffx.67513.1.S1_at	10.5	pectinesterase family protein
GmaAffx.57996.1.S1_at	10.4	pectinesterase family protein
GmaAffx.12889.1.S1_at	10.4	copper ion binding oxidoreductase
GmaAffx.43840.1.S1_at	10.4	No BLASTX match
GmaAffx.78316.1.S1_at	10.3	STP4 (SUGAR TRANSPORTER 4)
GmaAffx.63015.1.S1_at	10.3	invertase/pectin methylesterase inhibitor family protein
GmaAffx.46458.1.S1_s_at	10.2	hypothetical protein
GmaAffx.21929.1.S1_at	10.2	pectinesterase inhibitor
Gma.825.1.A1_at	10.2	pectinesterase family protein
GmaAffx.49287.1.A1_at	10.1	No BLASTX match
GmaAffx.34695.1.S1_at	10.1	AHA8 (ARABIDOPSIS H(+)-ATPASE 8)
GmaAffx.35627.1.A1_at	10.0	pectinesterase family protein
GmaAffx.84818.1.S1_at	10.0	leucine-rich repeat transmembrane protein kinase
GmaAffx.8097.1.S1_at	9.8	expressed protein
GmaAffx.53728.1.S1_at	9.8	beta-galactosidase
GmaAffx.85210.1.S1_at	9.7	SEC14 cytosolic factor family protein
GmaAffx.26070.1.S1_at	9.7	pollen Ole e 1 allergen and extensin family protein
GmaAffx.3553.1.S1_at	9.7	NOI (nitrate responsive protein)
Gma.1154.1.S1_at	9.6	No BLASTX match
GmaAffx.34708.1.S1_at	9.6	Hypothetical protein
GmaAffx.78349.1.S1_at	9.5	pollen specific phosphatase
GmaAffx.78268.1.S1_at	9.4	pectate lyase (Pollen-specific LAT 59)
Gma.15381.1.S1_at	9.3	senescence-associated protein
GmaAffx.89158.1.S1_at	9.3	SEC14 cytosolic factor

Meanwhile, 9 out of 30 highly abundant transcripts in mature soybean pollen are predicted to encode members of pectin esterase and pectate lyase families of cell-wall loosening enzymes (Table 1). Corresponding genes in *Arabidopsis *were among those with the highest expression in pollen [2,4,5]. It has been proposed that besides their possible involvement in pollen tube wall modification, these hydrolytic enzymes may be important for the penetration of the stigmatic tissues.

### Transcription factors up-regulated in the soybean pollen

A search using the matching AGI of the soybean probe set was performed at the *Arabidopsis *Gene Regulatory Information Server  to explore the different families of transcription factors represented by the up-regulated transcripts in the pollen to see which transcription factors might have a major role in regulating activities in the mature pollen. Although many of the transcripts are annotated as transcription factors, the corresponding Arabidopsis orthologues are yet to be grouped under the 50 different families at the AtTFDB collection and this is likely due to the lack of functional knowledge of the genes concerned. Nevertheless, at least 16 different families of transcription factors are represented as listed in Table 2.

**Table 2:** Family of transcription factors enriched in soybean pollen.

**Type of Transcription Factor**	**Number**
Zinc finger (C3H)	13
Zinc finer (C2H2)	12
MYB	3
bZIP	3
bHLH	3
NAC	2
PHD	2
WRKY	2
HSF	2
MADS	1
GRF	1
TUB	1
CCAAT-HAP3	1
Zinc finger (C2C2-CO-like)	1
AP2-EREBP	1
BBR/BPC	1

To investigate the different types of transcription factor families represented in the up-regulated transcripts in soybean pollen, a search using the matching AGI of the soybean probe set was performed at the *Arabidopsis *Gene Regulatory Information Server .

Zinc finger transcription factors are prominent in our differentially regulated gene data (25 genes). Although reported as pollen-specific genes in 1992 [11], zinc finger proteins act as master regulators (transcriptional repressors) in neuronal development, animal germ cells, and spermatogenesis [12]. For instance, Blimp1/Prdm1, a zinc finger transcriptional repressor, is the key regulator of early axis formation and primordial germ cell specification in animals [13]. Also, it has been shown that a targeted silencing of *Ovol1 *(also known as *movo1*), a zinc-finger transcription factor, leads to germ cell degeneration and defective sperm production in mice [14]. These proteins are also reported to be important regulatory molecules in various plant developmental processes, such as apical meristem development via chromatin remodeling process, anther development, and flowering.

It has been recently reported that a class of MYB factors regulate sperm cell formation in plants [15]. We identified three members of the MYB family as up-regulated in soybean pollen (Table 2). Certain MADS box proteins have been identified as pollen-specific in Antirrhinum [16] and have also been reported as an important non-classical transcriptional factor family in *Arabidopsis *pollen. Pina et al reported the over-representation of MADS box genes in the *Arabidopsis *pollen transcriptome, with 17 genes expressed in pollen and nine showing enrichment in pollen [3].

Plant homeodomain (PHD) finger transcription factors are up-regulated in soybean pollen. The PHD finger may promote both gene expression and repression through interactions with trimethylated lysine 4 on histone H3 (H3K4), a universal modification seen at the beginning of active genes [17,18]. PHDs are associated with chromatin condensation during mitosis or meiosis, general transcriptional machinery, and a transcriptional regulator required for proper development, flowering, and fertility of plants [19,20].

Meanwhile, very little is known about the physiological and developmental roles of WRKY proteins, another family of transcription factor up-regulated in the soybean pollen. Although the DNA binding site of WRKY proteins is well-defined, determining the individual role of WRKY factors remains a challenge [21,22]. Though the function of WRKY proteins in pollen is not clear, our data suggest an important and novel regulatory role for these proteins in soybean pollen.

A member of the basic helix-loop helix (bHLH) transcription factor also shows differential expression in soybean pollen; this group also shows a similar pattern of expression in *Arabidopsis *pollen. bHLH proteins are a family of transcription factors that bind to their DNA targets as dimmers [23,24]. They have been characterized in non-plant eukaryotes as important regulatory components in diverse biological processes such as the control of cell proliferation and the development of specific cell lineages. It has been shown that Tcfl5, a testis-specific bHLH protein, interacts with the regulatory region of the *Calmegin *gene promoter as a testis-specific activator of this gene and other testis-specific genes in mouse spermatogenesis [25]. Whether pollen-expressed bHLH transcription factors regulate sperm cell specific gene expression remains to be determined. Two NAC transcription factor family members are up-regulated in soybean pollen, suggesting a role of this family of proteins in the regulation of pollen genes, a function that to the best of our knowledge has not been reported for this class of genes.

### Transcripts associated with the ubiquitin system

Post-translational protein modifications play a critical role in most cellular processes through their unique ability to rapidly and reversibly alter the functions of synthesized proteins, multi-protein complexes, and intracellular structures. In eukaryotes, such modifications frequently occur by attaching a small polypeptide to the target protein. Ubiquitin and small ubiquitin-related modifiers (SUMO) are among those polypeptides [26]. Approximately 5% of *Arabidopsis *genes encode proteins that are predicted to be involved in the ubiquitin-proteasome system, and the regulation of protein degradation by ubiquitination is important in many plant processes [27]. Ubiquitin ligases that are associated with membrane-enclosed organelles are required for polarized pollen tube growth [28]. Furthermore, there has been a report of the enrichment of ubiquitin family genes in Arabidopsis sperm cells [8]. Our data contain many ubiquitin family genes, suggesting a role for this group of genes in pollen development through ubiquitin-mediated protein turnover (Table 3).

**Table 3:** Putative ubiquitin-related transcripts up-regulated in soybean pollen.

**Affymetrix Probe ID**	**Log_2 _Ratio**	**Annotation**
GmaAffx.33438.1.A1_at	4.8	ubiquitin-associated (UBA)/TS-N domain-containing protein
Gma.4406.3.A1_a_at	4.7	Probable ubiquitin-fold modifier 1 precursor (Protein PR46A)
GmaAffx.90938.1.S1_at	4.4	ubiquitin-protein ligase
Gma.17830.1.A1_at	4.1	ATUBP3 (UBIQUITIN-SPECIFIC PROTEASE 3)
GmaAffx.53665.1.S1_s_at	4.0	ubiquitin-protein ligase
GmaAffx.57775.1.S1_s_at	4.0	UBC10 (ubiquitin-conjugating enzyme 10)
Gma.3735.3.S1_at	3.3	ATUBC2 (UBIQUITING-CONJUGATING ENZYME 2); ubiquitin-protein ligase
Gma.5750.3.S1_a_at	3.3	UBP20 (UBIQUITIN-SPECIFIC PROTEASE 20)
GmaAffx.93424.1.S1_s_at	3.1	UBQ11 (UBIQUITIN 11)
Gma.8301.1.S1_a_at	2.9	MMZ1 (MMS ZWEI HOMOLOGE 1); ubiquitin-protein ligase
Gma.5750.1.S1_a_at	2.8	UBP20 (UBIQUITIN-SPECIFIC PROTEASE 20); DNA binding
Gma.10691.4.S1_s_at	2.6	UBC28; ubiquitin-protein ligase
GmaAffx.91367.1.S1_s_at	2.5	UBC10 (ubiquitin-conjugating enzyme 10); ubiquitin-protein ligase
GmaAffx.87774.1.S1_at	2.5	PRT1 (PROTEOLYSIS 1); ubiquitin-protein ligase
Gma.11119.5.S1_at	2.3	UBC9 (UBIQUITIN CONJUGATING ENZYME 9)
GmaAffx.65766.1.S1_at	2.2	Ubiquitin system component Cue
Gma.8301.3.S1_at	2.1	MMZ1 (MMS ZWEI HOMOLOGE 1); ubiquitin-protein ligase
Gma.10933.1.S1_a_at	2.1	UBQ13 (ubiquitin 13)
Gma.10435.1.S1_at	2.1	UBC32 (ubiquitin-conjugating enzyme 31)
Gma.11119.4.S1_at	2.0	UBC10 (ubiquitin-conjugating enzyme 10)
GmaAffx.55629.1.S1_at	1.9	UBP25 (UBIQUITIN-SPECIFIC PROTEASE 25)
GmaAffx.50783.1.S1_at	1.9	SKIP6 (SKP1 INTERACTING PARTNER 6); ubiquitin-protein ligase
Gma.5718.1.A1_s_at	1.8	UBC22 (ubiquitin-conjugating enzyme 18); ubiquitin-protein ligase

### Signal transduction and transporters

Approximately, 100 different signalling proteins, such as 14-3-3 proteins and kinases are up-regulated at the gene level in the soybean pollen. 14-3-3 proteins are among the most important and versatile proteins in eukaryotes [29]. They interact with many regulatory proteins like transcription factors (by protein-protein interaction) and alter their activity, in addition to performing regulatory roles by shuttling proteins between various cellular locations. In plants, it has been reported that 14-3-3 proteins regulate the H-ATPase pumps of the plasma membrane [30]. As expected, calcium-related proteins are enriched in soybean pollen, as they are important regulators of pollen germination and tube growth. Calcium and calcium sensor proteins such as calmodulin (CaM), a universal calcium sensor protein, play important roles in gene regulation, and hence plant growth and development [31,32]. It has been shown that calcium transporters are key regulators of pollen tube development and fertilization in flowering plants [33]. In addition, CaM binding proteins, such as maize pollen calmodulin-binding protein (MPCBP) and NPG1 (*no pollen germination1*) in *Arabidopsis*, are specifically expressed in pollen and regulate pollen germination, as supported by the observation that down-regulation of these genes resulted in the inability of the pollen to germinate [34,35]. As expected, we identified many calcium-related genes in our soybean dataset (Table 4). Some of these proteins are already known to be pollen-specific, and many are highly up-regulated (up to 256-fold) as compared to sporophytic tissues, highlighting the importance of these proteins in pollen biology.

**Table 4:** Representative transcripts under the functional category of signal transduction with higher expression level in the soybean pollen in comparison to the sporophytic tissues.

**Affymetrix Probe ID**	**Log_2 _Ratio**	**Annotation**
GmaAffx.43921.1.S1_at	9.1	Putative calcium-dependent protein kinase
GmaAffx.80188.1.A1_at	8.9	Calcium-dependent calmodulin-independent protein kinase isoform 2
GmaAffx.55767.1.S1_at	8.5	calcium-binding protein
GmaAffx.9280.1.S1_at	7.7	CPK7 (CALMODULIN-DOMAIN PROTEIN KINASE 7)
Gma.15500.1.S1_at	6.9	calcium ion binding protein
Gma.15500.2.A1_at	6.6	Calcium-binding EF-hand
GmaAffx.89567.1.A1_at	6.5	CPK1 (calcium-dependent protein kinase isoform AK1)
GmaAffx.43741.1.S1_at	5.7	calcium ion binding protein
Gma.8417.1.S1_at	5.6	CPK4 (calcium-dependent protein kinase 4)
GmaAffx.23909.1.S1_at	5.2	CPK28 (calcium-dependent protein kinase 28)
GmaAffx.89301.1.A1_at	3.9	Calcium-dependent protein kinase CDPK1444
GmaAffx.92868.1.S1_s_at	3.4	CAM7 (CALMODULIN 7); calcium ion binding

Transport proteins, including membrane pumps, represent one of the largest up-regulated gene sets in the soybean pollen (Additional File 1). Table 5 shows a representative list of transcripts classified under the functional category of "transporter" and this includes those predicted to encode SUGAR TRANSPORTER 4 (STP4), ARABIDOPSIS H(+)-ATPASE 8 (AHA8), AHA9, monosaccharide/H+ symporter (STP), amino acid transporter, Ca^2+ ^pumps and a putative phosphate translocator. Similar categories of transcripts have been reported to be up-regulated in *Arabidopsis *pollen [36].

**Table 5:** Representative up-regulated transcripts in the soybean pollen under the functional category of transporter

**Affymetrix Probe ID**	**Log_2 _Ratio**	**Annotation**
GmaAffx.78316.1.S1_at	10.3	STP4 (SUGAR TRANSPORTER 4); carbohydrate transporter/sugar porter
GmaAffx.34695.1.S1_at	10.1	AHA8 (ARABIDOPSIS H(+)-ATPASE 8); ATPase
Gma.18042.1.S1_at	6.4	mitochondrial substrate carrier family protein
GmaAffx.43336.1.S1_at	6.3	SIP2;1 (SMALL AND BASIC INTRINSIC PROTEIN 2); transporter
Gma.14613.1.A1_at	6.2	kelch repeat-containing F-box family protein
Gma.3527.1.S1_at	5.8	calcium-transporting ATPase
Gma.14065.1.A1_at	5.8	membrane protein-related
Gma.4648.1.S1_at	5.6	permease-related proetin
GmaAffx.18381.1.S1_at	5.6	mitochondrial substrate carrier family protein
GmaAffx.75679.1.S1_at	5.5	magnesium transporter CorA-like family protein (MRS2-2)
GmaAffx.5958.1.A1_at	5.2	AAP3 (amino acid permease 3); amino acid permease
GmaAffx.66056.1.S1_at	5.0	AHA9 (Arabidopsis H(+)-ATPase 9)
GmaAffx.79100.1.S1_at	4.8	PPI1 (PROTON PUMP INTERACTOR 1)
GmaAffx.35242.1.S1_at	4.8	haloacid dehalogenase-like hydrolase family protein
GmaAffx.40934.1.S1_at	4.7	SKOR (stelar K+ outward rectifier); cyclic nucleotide binding/outward rectifier potassium channel
GmaAffx.55782.1.S1_at	4.4	PIP2;4/PIP2F (plasma membrane intrinsic protein 2;4)
GmaAffx.50740.1.S1_at	4.4	ATOPT1 (oligopeptide transporter 1)
Gma.7510.1.A1_at	4.3	sodium proton exchanger, putative (NHX6)
Gma.16713.2.S1_a_at	4.2	mitochondrial substrate carrier family protein
GmaAffx.22309.1.S1_at	4.2	magnesium transporter CorA-like family protein
GmaAffx.86058.1.S1_at	4.1	outward rectifier potassium channel
Gma.18090.1.S1_at	4.1	PPI1 (PROTON PUMP INTERACTOR 1)
Gma.14250.1.S1_at	3.6	amino acid transporter family protein
GmaAffx.45132.1.S1_at	3.6	PGP9 (P-GLYCOPROTEIN 9) ATPase
GmaAffx.28163.1.S1_at	3.6	mitochondrial substrate carrier family protein
GmaAffx.73726.1.S1_at	3.3	nucleobase:cation symporter
GmaAffx.38263.1.S1_at	3.3	outer membrane OMP85 family protein
Gma.11250.3.S1_a_at	3.3	magnesium transporter CorA-like family protein (MRS2-1)
Gma.3044.2.S1_s_at	3.1	PPI1 (PROTON PUMP INTERACTOR 1)
Gma.17362.1.S1_at	3.0	potassium channel tetramerisation domain-containing protein
GmaAffx.58615.1.S1_at	2.9	sodium proton exchanger, putative (NHX6)
GmaAffx.11496.1.S1_at	2.9	SULTR3;5 (SULTR3;5)
GmaAffx.84719.1.S1_at	2.8	phosphate translocator-related
Gma.3044.1.S1_at	2.8	PPI1 (PROTON PUMP INTERACTOR 1)
GmaAffx.5951.1.S1_at	2.7	metal transporter family protein
Gma.2760.1.S1_at	2.0	SULTR4;2 (sulfate transporter 4;2); sulfate transporter
Gma.17298.2.S1_a_at	1.9	integral membrane transporter family protein
Gma.13872.1.S1_at	1.8	sugar transporter, putative

Higher plants possess two distinct families of sugar carriers: the disaccharide transporters that primarily catalyse sucrose transport and the monosaccharide transporters that mediate the transport of a variable range of monosaccharides [37]. The *STP4 *gene encodes a membrane located monosachharide H+ symporter that can catalyze the uptake of various monosaccharides [38]. High expression of monosachharide transporter in soybean pollen points towards glucose and fructose as preferred source of nutrition for pollen germination and tube growth. A similar pollen specific expression of a putative hexose transporter gene was reported in *Arabidopsis *and *Petunia *[39,40]. It has been proposed that in species where monosachharides are taken up preferentially, sucrose might be hydrolysed to glucose and fructose by a cell-wall invertase before uptake by monosachharide transporters in the growing pollen tube.

High up-regulation of H^+^ATPases including those encoding AHA8 and AHA9 in soybean pollen points to an essential role similar to their *Arabidopsis *and *Nicotiana *counterparts. The expression of AHA8 and AHA9 has been shown to be pollen-specific in *Arabidopsis *[3]. Recently, a pollen H+ ATPases has been shown to be associated with the tip growth in Nicotiana pollen tubes [41]. Uptake and translocation of cationic nutrients play essential roles in plant growth, nutrition, signal transduction, and development [42]. The plant cation transporter gene families include potassium transporters and channels, sodium transporters, calcium antiporters, cyclic nucleotide-gated channels and cation diffusion facilitator proteins. Our data show that several of the members of cation/proton exchanger family proteins are expressed at a higher level in the soybean pollen in comparison to those of sporophytic tissues. Bock et al [36] reported that fourteen members of the cation/proton exchanger (CHX) gene family are expressed late in pollen development and also raised questions about their roles and multiplicity. The possibility that they are localized to different intracellular compartments was proposed. It is noteworthy that a similar multiplicity of cation/proton exchanger family genes that are up-regulated in the soybean pollen is apparent in our data.

### WD-40 repeat proteins

WD-40 repeat proteins are defined by the presence of four or more repeating units containing a conserved core of approximately 40 amino acids that usually end with tryptophan-aspartic acid (WD). WD-repeat proteins are conserved in animals and plants, where they participate in complexes involved in chromatin metabolism and gene expression [43-45]. They also have been reported to be transcriptional repressors that interact either with co-repressors or in a complex with histone deacetylases, to regulate spermatogenesis, and to function as mitotic checkpoints to ensure accurate chromosome segregation. A number of WD-repeat protein are up-regulated in the soybean pollen (Table 6) implicating their likely involvement in regulating pollen development.

**Table 6:** Putative WD-40 repeats protein up-regulated in the soybean pollen in comparison to the sporophytic tissues.

**Affymetrix Probe ID**	**Log_2 _Ratio**	**Annotation**
Gma.15007.3.S1_s_at	2.7	transducin family protein/WD-40 repeat family protein
GmaAffx.1810.1.A1_at	2.1	transducin family protein/WD-40 repeat family protein
GmaAffx.34663.1.A1_at	6.8	transducin family protein/WD-40 repeat family protein
GmaAffx.52213.1.S1_at	3.3	WD-40 repeat family protein
Gma.15213.1.S1_at	2.9	WD-40 repeat family protein
Gma.15617.2.S1_at	2.6	WD-40 repeat family protein/beige-related
Gma.7017.2.S1_s_at	2.5	transducin family protein/WD-40 repeat family protein
GmaAffx.30148.1.S1_at	2.6	WD-40 repeat family protein/beige-related
GmaAffx.78269.1.S1_at	2.2	WD-40 repeat family protein/beige-related
GmaAffx.5267.1.S1_at	4.8	WD-40 repeat family protein/zfwd2 protein (ZFWD2), putative
GmaAffx.91103.1.S1_at	2.4	transducin family protein/WD-40 repeat family protein
GmaAffx.35843.1.S1_at	2.1	WD40-like protein

### Heat shock proteins

Heat shock proteins (HSPs)/chaperones) are divided in five major families: the HSP70, the HSP60, the HSP 90, the HSP 100 families and a small HSP family [46]. The accumulation of heat shock proteins (HSPs) under heat and other abiotic stresses has been suggested to play a key role in the acquisition of thermotolerance in plants and other organisms. At the cell level these proteins are responsible for protein folding, assembly, and translocation, and can assist in protein re-folding under stress conditions. Some studies could not detect heat shock response in developing microspores or mature pollen of various species [47,48] while others have shown that many HSPs are expressed in microspores and mature pollen [49].

**Figure 3:**
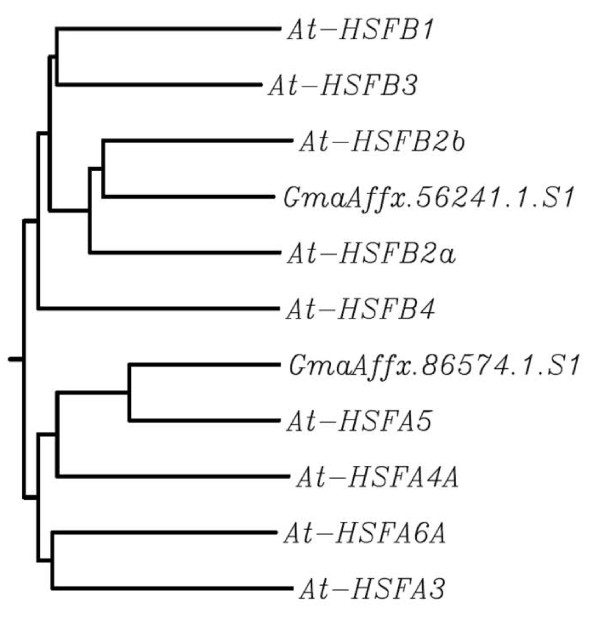
**Phylogenetic relationship of virtually translated GmaAffx.56241.1.S1 and GmaAffx.86574.1.S1 with heat shock factors (At-HSF) from *Arabidopsis thaliana***. The phylogenetic tree is constructed using CLUSTAL W (version 1.83) and the results displayed as NJ-tree with branch length. Protein sequences of At-HSFs were retrieved from TAIR website  and the predicted protein sequence for GmaAffx.56241.1.S1 or GmaAffx.86574.1.S1 from PHYTOZOME .

It is interesting to note that in our present study on mature soybean pollen transcriptome, there is significant up-regulation of transcripts encoding heat shock proteins as well as heat shock transcription factors HSFB2A and HSFA5 (Table 7; Figure 3). A recent study on transcriptome changes during pollen germination showed significant up-regulation of HSPs during pollen germination and tube growth, and many of these HSPs are undetectable at the expression level in mature pollen [50]. These authors proposed that these HSPs might function as molecular chaperones for protein modification processes during pollen germination and tube growth. Heat shock factors are the primary molecules responsible for activating genes responsive to both heat stress and other stressors [51]. The up-regulation of heat shock transcription factor HSFB2A and HSFA5 in soybean pollen matches similar up regulation of its counterpart in *Arabidopsis *pollen [51]. The plant HSF family has been reported to comprise more than 20 members with recent evidence pointing towards the unique functions of individual HSFs in signal transduction pathways activated in response to environmental stress and during development. Conserved up-regulation of HSFB2A and HSFA5 in both soybean and Arabidopsis pollen points towards unique role of these transcription factors in pollen development and possibly in gamete development. It is interesting to note that heat shock proteins are known for their role in animal spermatogenesis by acting as molecular chaperones to assist with protein folding [52].

**Table 7:** Up-regulated transcripts in the soybean pollen predicted to encode heat shock-related proteins.

**Affymetrix Probe ID**	**AtGI**	**Annotation**	**Log2 Ratio**
GmaAffx.87467.1.S1_at	AT1G52560	small heat shock protein-like (HSP26.5-P)	3.1
Gma.11105.1.A1_at	AT1G61770	DNAJ heat shock N-terminal domain-containing protein	3.1
GmaAffx.25874.1.S1_at	AT2G25560	DNAJ heat shock N-terminal domain-containing protein	3.3
GmaAffx.85437.1.S1_at	AT2G26890	GRV2 (KATAMARI2); binding/heat shock protein binding	2.8
Gma.17947.1.S1_at	AT2G29500	17.6 kDa class I small heat shock protein (HSP17.6B-CI)	4.4
GmaAffx.93268.1.S1_at	AT2G29500	17.6 kDa class I small heat shock protein (HSP17.6B-CI)	4
GmaAffx.57556.1.S1_at	AT3G08970	DNAJ heat shock N-terminal domain-containing protein	3.6
GmaAffx.69311.1.S1_at	AT3G46230	ATHSP17.4 (A. thaliana heat shock protein 17.4)	4.1
Gma.3422.1.S1_at	AT4G13830	J20 (DNAJ-LIKE 20); heat shock protein binding	3.6
GmaAffx.86574.1.S1_at	AT4G13980	AT-HSFA5 (heat shock transcription factor A5)	2.0
GmaAffx.9985.1.S1_at	AT5G06410	DNAJ heat shock N-terminal domain-containing protein	2.4
GmaAffx.69544.1.S1_s_at	AT5G12020	17.6 kDa class II heat shock protein (HSP17.6-CII)	3.9
GmaAffx.69544.1.S1_at	AT5G12020	17.6 kDa class II heat shock protein (HSP17.6-CII)	3.7
Gma.7766.1.S1_at	AT5G12020	17.6 kDa class II heat shock protein (HSP17.6-CII)	3.6
GmaAffx.69544.2.S1_at	AT5G12020	17.6 kDa class II heat shock protein (HSP17.6-CII)	2.5
GmaAffx.56241.2.S1_at	AT5G62020	AT-HSFB2A (heat shock transcription factor B2A)	6.5
GmaAffx.56241.1.S1_at	AT5G62020	AT-HSFB2A (heat shock transcription factor B2A)	4.3

## Conclusion

This is the first report on transcriptional profiling of the pollen of a major legume crop. The current knowledge from pollen transcriptome profiling with microarrays is limited to the model plant, *Arabidopsis*. Our data will extend the current understanding of pollen biology and gene regulation by providing a set of robustly selected, differentially expressed genes in soybean pollen. We also provide a number of genes with unknown functions that are highly expressed in the pollen and could be tested in many functional analyses to increase our understanding of gene regulation in pollen. Most of the genes important for sporophytic organs are highly repressed in pollen. Regulation of these genes is probably controlled at the transcriptional level by transcriptional factors and chromatin remodelling machinery, as pollen contains a variety of transcription factor transcripts for use in different developmental situations. Further research on the candidates reported in this study should provide new insights into the understanding of plant male gametophyte development other than the current knowledge provided by research on model plants.

## Methods

### Plant growth and pollen collection

**Figure 4:**
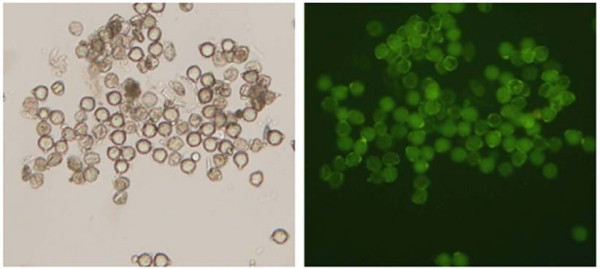
**Photomicrograph of isolated pollen under light microscopy (left) and fluorescein diacetate viability screen in epifluorescence microscopy (right)**.

Soybean plants [*Glycine max*. (L) Merr. Cv. Bragg] were used in this study. The plants used for pollen collection were grown in a temperature-controlled greenhouse with a 16 hour light/8 hour dark photoperiod at 30°C. They were grown in vermiculite with the addition of a slow release fertilizer (osmocote). When the plants had matured and developed significant biomass, flowering was induced by changing the photoperiod to 12 hours. Pollen was collected on coverslips by rubbing isolated anthers together, and anther tissue was removed from the coverslip prior to freezing at -80°C. Pollen purity and viability was assessed by microscopic observations and fluorescein diacetate test (Figure 4).

### RNA isolation and microarray hybridization

Total RNA from pollen or sporophytic tissues (primary stem, primary roots and mature leaves of 10-day-old soybean seedlings) was isolated using the QIAGEN RNeasy Mini Kit (QIAGEN) and eluted with nuclease-free water. Subsequent cDNA labelling and Affymetrix Soybean GeneChip hybridization was carried out by AGRF (Australian Genome Research Facility, Melbourne, Australia) using 3 μg of total RNA according to protocols outlined in .

### Analysis of expression data

The GeneChip^® ^Soybean Genome Array (Affymetrix, Inc.) containing probe sets for 37,500 transcripts was used in this study. Three biological replicates for pollen and two biological replicates for sporophytic tissues were used. Raw numeric values representing the signal of each feature were imported into AffylmGUI (Affymetrix linear modeling Graphical User Interface [7] that uses the Empirical Bayes linear modeling approach of Smyth (2005)[53] for identifying differentially expressed genes in pollen. The data were normalized using Robust Multiarray Averaging (RMA) method and a linear model was then used to average data between replicate arrays and to look for variability between them [7]. The list of transcripts that were detected to be differentially expressed at adjusted p-value of < 0.05 were used for all subsequent analysis. All microarray data have been submitted to Gene Expression Omnibus (GEO) at NCBI  under the accession GSE 12286.

To obtain the number of pollen-expressed genes (expressed in pollen and sporophytic tissues), we collect the expression signals, average expression values, and present/absent calls from AffylmGUI (RMA data) and sorted the data in Excel. To find pollen-specific group of genes, we used the following criteria: 1) showed statistically significant differential expression at adjusted *p*value < 0.05; 2) possessed a signal greater than or equal to 100 on each replicate; 3) had a cut-off value of a 2-fold change; and 4) had "Absence" calls on all of the sporophytic replicates.

The annotation for the transcripts represented by the soybean GeneChip^® ^was downloaded from the Seed Development website . The annotation is based on the best BLASTX match of the corresponding soybean sequences against TAIR Arabidopsis protein database or NCBI non-redundant protein database (expect value < 0.01). Functional categories for these transcripts were assigned based on the EU *Arabidopsis *sequencing project [54] as described at the Seed Development website .

## Authors' contributions

FH carried out the RNA extractions, participated in the microarray experiment and drafted the manuscript. CEW was responsible for the organization of the data and manuscript editing. PG was responsible for organizing flowering soybean plants and collecting pollen. PLB and MBS were responsible for the design of the project, overall coordination of experiments and manuscript editing. All authors read and approved the final manuscript.

## Supplementary Material

Additional file 1 **Transcripts identified to be up-regulated in the soybean mature pollen in comparison to sporophytic tissues**. The spreadsheet contains all the transcripts that are identified to be up-regulated in the soybean mature pollen in comparison to sporophytic tissues Click here for file

Additional file 2 **Transcripts identified to be down-regulated in the soybean mature pollen in comparison to sporophytic tissues**. The spreadsheet contains all the transcripts that are identified to be down-regulated in the soybean mature pollen in comparison to sporophytic tissues Click here for file
